# Disruptions in Care for Medicare Beneficiaries With Severe Mental Illness During the COVID-19 Pandemic

**DOI:** 10.1001/jamanetworkopen.2021.45677

**Published:** 2022-01-28

**Authors:** Alisa B. Busch, Haiden A. Huskamp, Pushpa Raja, Sherri Rose, Ateev Mehrotra

**Affiliations:** 1 McLean Hospital, Belmont, Massachusetts; 2 Department of Health Care Policy, Harvard Medical School, Boston, Massachusetts; 3 US Department of Veterans Affairs Greater Los Angeles Medical Center, Los Angeles, California; 4 Center for Health Policy and Center for Primary Care and Outcomes Research, Stanford University, Stanford, California; 5 Beth Israel Deaconess Medical Center, Boston, Massachusetts

## Abstract

**Importance:**

Little is known about changes in care for individuals with severe mental illness during the COVID-19 pandemic.

**Objective:**

To examine changes in mental health care during the pandemic and the use of telemedicine in outpatient care among Medicare beneficiaries with severe mental illness.

**Design, Setting, and Participants:**

This population-based cohort study included Medicare beneficiaries (age ≥18 years) diagnosed with schizophrenia and schizophrenia-related disorders or bipolar I disorder. Care patterns during January to September 2020 for a cohort defined in 2019 were compared with those during January to September 2019 for a cohort defined in 2018.

**Exposures:**

Start of COVID-19 pandemic in the United States, defined as week 12 of 2020.

**Main Outcomes and Measures:**

Use of mental health–related outpatient visits, emergency department visits, inpatient care, and oral prescription fills for antipsychotics and mood stabilizers during 4-week intervals. Multivariable logistic regression analyses examined whether the pandemic was associated with differential changes in outpatient care across patient characteristics.

**Results:**

The 2019 cohort of 686 214 individuals included 389 245 (53.8%) women, 114 073 (15.8%) Black and 526 301 (72.8%) White individuals, and 477 353 individuals (66.0%) younger than 65 years; the 2020 cohort of 723 045 individuals included 367 140 (53.5%) women, 106 699 (15.6%) Black and 497 885 (72.6%) White individuals, and 442 645 individuals (64.5%) younger than 65 years. Compared with 2019, there were large decreases during the pandemic’s first month (calendar weeks 12-15) in individuals with outpatient visits (265 169 [36.7%] vs 200 590 [29.2%]; 20.3% decrease), with antipsychotic and mood stabilizer medication prescription fills (216 468 [29.9%] vs 163 796 [23.9%]; 20.3% decrease), with emergency department visits (12 383 [1.7%] vs 8503 [1.2%]; 27.7% decrease), and with hospital admissions (11 564 [1.6%] vs 7912 [1.2%]; 27.9% decrease). By weeks 32 to 35 of 2020, utilization rebounded but remained lower than in 2019, ranging from a relative decrease of 2.5% (outpatient visits) to 12.9% (admissions). During the full pandemic period (weeks 12-39) in 2020, 1 556 403 of 2 743 553 outpatient visits (56.7%) were provided via telemedicine. In multivariable analyses, outpatient visit use during weeks 12 to 25 of 2020 was lower among those with disability (odds ratio, 0.95; 95% CI, 0.93-0.96), and during weeks 26 to 39 of 2020, it was lower among Black vs non-Hispanic White individuals (OR, 0.97; 95% CI, 0.95-0.99) and those with dual Medicaid eligibility (OR, 0.96; 95% CI, 0.95-0.98).

**Conclusions and Relevance:**

In this cohort study, despite greater use of telemedicine, individuals with severe mental illness experienced large disruptions in care early in the pandemic. These narrowed but persisted through September 2020. Disruptions were greater for several disadvantaged populations.

## Key Points

**Question:**

Was the COVID-19 pandemic associated with changes in mental health–related utilization for individuals with severe mental illness in the United States?

**Findings:**

In this cohort study of more than 650 000 Medicare beneficiaries with schizophrenia or bipolar I disorders, there were statistically significant decreases in mental health–related outpatient, emergency department, and inpatient use as well as medication fills, particularly early in the pandemic, despite widespread use of telemedicine. Statistically significant differentially greater decreases in outpatient utilization were observed among individuals who were Black, had dual Medicare-Medicaid eligibility, or had disability.

**Meaning:**

In this study, individuals with severe mental illness experienced substantial disruptions in care during the pandemic, and these disruptions were greater among disadvantaged populations.

## Teaser

This cohort study examines changes in mental health care during the pandemic and the use of telemedicine in outpatient care among Medicare beneficiaries with severe mental illness.

## Introduction

Individuals with a severe mental illness, such schizophrenia or bipolar I disorder, may be particularly vulnerable to care disruptions during the ongoing COVID-19 pandemic. They frequently are marginalized, have disability, are unhoused, live in poverty, and are more socially isolated.^1,2^ While telemedicine use increased in the pandemic and is feasible in treating psychotic disorders,^3,4^ some individuals with severe mental illness lack the tools or digital literacy to use telemedicine.^5,6,7^ Additionally, they have higher rates of chronic medical conditions that are associated with greater risk of COVID-19 morbidity and mortality^8,9,10^—such as diabetes, cardiovascular disease, hypertension, and chronic lung disorders^11,12^—and therefore may be more reluctant to seek in-person care. Together, these factors may cause some to forgo needed outpatient care which in turn could result in greater use of emergency care.^13,14,15^ Additionally, the pandemic may exacerbate preexisting disparities among individuals who belong to racial or ethnic minority groups or rural residents.^16,17,18,19,20,21,22,23^

To our knowledge, prior research describing care utilization among individuals with severe mental illness during the pandemic is limited to 1 study of a US regional community health center population through May 2020.^24^ We extend this research by examining trends in a national sample of adult Medicare beneficiaries through September 2020. A large proportion of individuals with severe mental illness have Medicare insurance,^25^ specifically those who are aged 65 years or older and those who are disabled from their illness. Our focus was on outpatient care and whether any changes in outpatient visits were greater in disadvantaged populations, ie, those with disability, those with low income, those who belong to racial and ethnic minority groups, and those who reside in rural locations; we also examine changes in acute care visits, medication fills, and the extent to which telemedicine was used in outpatient care.

## Methods

The Harvard Medical School institutional review board exempted this study from review and the requirement for informed consent because the data were secondary and deidentified. The study followed the Strengthening the Reporting of Observational Studies in Epidemiology (STROBE) guideline for cohort studies.^26^

### Study Cohorts

Using claims from all Medicare beneficiaries aged 18 years and older between January 2018 and September 2020, we compared utilization in the first 9 months of 2020 (the pandemic year) among a cohort of individuals with severe mental illness with utilization in the first 9 months of 2019 among a similar cohort. Each cohort was defined based on utilization in the prior calendar year (eg, the 2020 cohort was defined in 2019). Cohort members were required to have at least 21 months continuous enrollment in the fee-for-service Medicare program: 12 months in the first year and 9 months in the subsequent year (ie, the 2019 cohort had to have been enrolled from January 1, 2018, to September 30, 2019; the 2020 cohort, January 1, 2019, to September 30, 2020). The cohorts were not mutually exclusive.

Consistent with prior literature,^18,27,28,29^ individuals were defined as having schizophrenia or related disorders if they had at least 1 schizophrenia or related disorder hospitalization (*International Statistical Classification of Diseases and Related Health Problems, Tenth Revision *[*ICD-10*] codes F20-F29) or at least 2 outpatient visits on different service dates where the primary or secondary diagnosis on the claim was for schizophrenia or related disorders. Among those not defined as having schizophrenia, individuals were defined as having bipolar I disorder (*ICD-10* codes F30, F31.0-F31.7, and F31.9) using the same hospitalization and visit requirements.

### Outcomes

We examined the use of mental health (1) outpatient services, (2) emergency department (ED) services, (3) hospital admissions, and (4) antipsychotic or mood stabilizer medication prescription fills in the study cohorts. Mental health care was defined as care for mental health or substance use disorders (*ICD-10* codes F10-F16, F17-F69, F80-F89, or F90-F99). Outpatient mental health visits were those for which a mental health or substance use disorder diagnosis was the primary or secondary reason (eTable 1 in the Supplement). We counted a maximum of 1 visit per day. Among outpatient visits, we also examined the fraction delivered via telemedicine (eTable 1 in the Supplement). Mental health ED visits were defined as visits with the primary diagnosis of a mental health or substance use disorder or self-injury in any diagnosis field^30^ that did not result in a hospitalization. Mental health hospitalizations were those with the primary admission reason of a mental health or substance use disorder or with self-injury in the primary or secondary diagnosis field (eTable 2 in the Supplement). We excluded hospital admissions in September because inpatient data are submitted to Medicare on discharge and therefore may be incomplete. If in a single day a beneficiary had a mental health visit from more than 1 level of care (eg, ED and outpatient), the higher intensity service encounter was selected.

The medication outcome was defined as prescription fills of oral first- or second-generation antipsychotics, lithium, or anticonvulsants approved by the US Food and Drug Administration or guideline recommended for the treatment of bipolar disorder (valproic acid, carbamazepine, or lamotrigine). In a given 4-week interval, if a patient had a prescription fill for 1 to 30 days’ supply of at least 1 (or more) of these medications, then we defined this as a medication fill for that interval. If they had 31 to 60 days’ supply, then the supply counted as a medication fill for two 4-week intervals (this interval and the subsequent), and 61 to 90 days’ supply was counted as supply for three 4-week intervals.

### Demographic and Clinical Characteristics

Demographic characteristics included documented sex (male, female), age group (18-24, 25-34, 35-44, 45-54, 55-64, 65-74, 75-84, and >84 years), race and ethnicity documented in the Medicare enrollment file (Black, Hispanic, White, other [Medicare categories: Asian, American Indian or Alaska Native, and other], and unknown), census region, Medicaid dual eligibility (denoting patients with lower income), disability status, and whether the beneficiary resided in a rural area consistent with Medicare’s definition.^31^ Race and ethnicity were included because prior research documents preexisting disparities among individuals who belong to minoritized racial and ethnic groups in receipt of and access to mental health care. Clinical characteristics included co-occurring substance use disorder (defined as at least 2 visits on different service dates in year with a substance use disorder diagnosis code in any diagnostic field), potentially more severe or unstable severe mental illness (defined as whether a beneficiary had at least 1 mental health hospitalization in the cohort-defining year), and medical comorbidities associated with greater morbidity and mortality among individuals with COVID-19 (cardiovascular conditions, diabetes, hypertension, or chronic lung conditions)^8,9^ (eTable 2 in the Supplement).

### Statistical Analysis

We first described the weekly percentage of outpatient visits that were delivered via telemedicine (video, audio only, or either) in 2020. In bivariate analyses using χ^2^ tests, we compared the percentage of patients in 2019 and 2020 who had at least 1 mental health: (1) outpatient visit (any type and by subtypes: individual or family therapy, group therapy, and electroconvulsive therapy), (2) ED visit, (3) hospital admission, and (4) antipsychotic and mood stabilizer prescription fill in 4-week intervals beginning January 2019 and January 2020. For outpatient visits, we also examined the percentage who had a visit in 3- and 6-month intervals since the pandemic started (weeks 12-25, 26-39, and 12-39 of 2020). For each of these intervals, we estimated 95% CIs of the percentage-point difference between 2020 and 2019. Our outcomes start with utilization in week 12 because the US national declaration of emergency began on March 13th, 2020, the end of the 11th week that year. We excluded week 40 because it had an unequal number of days in 2019 vs 2020.

We fit multivariable logistic regression models examining the likelihood of having any outpatient mental health visit in calendar periods that corresponded to the 3- and 6-month intervals described previously. To examine whether changes varied across demographic characteristics that are associated with known disparities, digital access difficulties, comorbidities associated with increased risk of COVID-19 mortality, or more complex or severe/unstable psychiatric illnesses, we included interactions with year in the regression for the following patient characteristics: race and ethnicity, rurality,^16,17,18,19,20,21,22,23^ dual eligibility, disability,^5,6^ co-occurring substance use disorder, mental health hospitalization in the prior year, and medical comorbidities.^8,9^ Because 54.8% of the 2019 and 2020 cohorts overlapped (ie, beneficiaries were present in both cohort years), we clustered standard errors by patient.

Analyses were conducted in SAS version 7.15 (SAS Institute). Statistical significance was set at *P* < .05, and regression tests were 2-tailed.

## Results

The 2019 cohort had 723 045 beneficiaries, and the 2020 cohort had 686 214 (Table 1; eFigure 1 in the Supplement). The cohorts were similar in the observed demographic and clinical characteristics: nearly two-thirds were younger than 65 years (477 353 [66.0%] in 2019; 442 645 [64.5%] in 2020), approximately half were female (389 245 [53.8%] in 2019; 367 140 [53.5%] in 2020), and nearly three-quarters White (526 301 [72.8%] in 2019; 497 885 [72.6%] in 2020), with next largest group being Black (approximately 16%: 114 073 [15.8%] in 2019; 106 699 [15.6%] in 2020). More than one-fourth were rural residents. Most had disability (591 346 [81.8%] in 2019; 556 999 [81.2%] in 2020) and were dually eligible for Medicaid (approximately 78%).

**Table 1.** Characteristics of Study Cohorts of Medicare Beneficiaries With Severe Mental Illness^a^

Characteristic	Participants, No. (%)	
	2019 cohort (n = 723 045)	2020 cohort (n = 686 214)
Demographic characteristics		
Sex		
Female	389 245 (53.8)	367 140 (53.5)
Male	333 800 (46.2)	319 073 (46.5)
Age, y		
18-24	6044 (0.8)	5903 (0.9)
25-34	54 205 (7.5)	51 071 (7.4)
35-44	101 928 (14.1)	96 156 (14.0)
45-54	139 491 (19.3)	125 027 (18.2)
55-64	175 685 (24.3)	164 497 (24.0)
65-74	151 377 (20.9)	152 615 (22.2)
75-84	67 180 (9.3)	66 214 (9.7)
>84	27 135 (3.8)	24 731 (3.6)
Race and ethnicity^b^		
African American or Black	114 073 (15.8)	106 699 (15.6)
Hispanic or Latinx	50 508 (7.0)	48 743 (7.1)
White	526 301 (72.8)	497 885 (72.6)
Other	24 126 (3.3)	23 567 (3.4)
Unknown	8037 (1.1)	9320 (1.4)
Census region		
Northeast	161 795 (22.4)	153 380 (22.4)
Midwest	181 172 (25.1)	170 835 (24.9)
West	122 026 (16.9)	119 271 (17.5)
South	257 848 (35.7)	242 061 (35.3)
Other^c^	204 (0.03)	217 (0.03)
Rural resident	188 096 (26.0)	176 842 (25.8)
Disability	591 346 (81.8)	556 999 (81.2)
Dually eligible for Medicaid	566 938(78.4)	530 744 (77.3)
Clinical and utilization characteristics		
Diagnosis		
Schizophrenia or related disorders	428 622 (59.3)	407 297 (59.4)
Bipolar I	294 423 (40.7)	278 917 (40.7)
Co-occurring substance use disorder	65 306 (9.0)	61 611 (9.0)
≥1 Medical comorbidity associated with higher COVID-19 risk^d^	279 020 (38.6)	258 572 (37.7)
Any mental health hospitalization in prior year	105 265 (14.6)	96 528 (14.1)

^a^ Severe mental illness defined as schizophrenia and related disorders or bipolar I disorder. All characteristics (demographic and clinical/utilization) established in the cohort year. That is, for the 2019 cohort, they were established in 2018 and, for the 2020 cohort, in 2019.

^b^ As documented in Medicare enrollment file. Other indicates Asian, American Indian or Alaska Native, and other.

^c^ Guam, Puerto Rico, and the Virgin Islands.

^d^ Diagnosis of at least 1 of the following: cardiovascular disorder, diabetes, hypertension, chronic lung disorder.

### Outpatient Telemedicine Use

**Figure 1.**
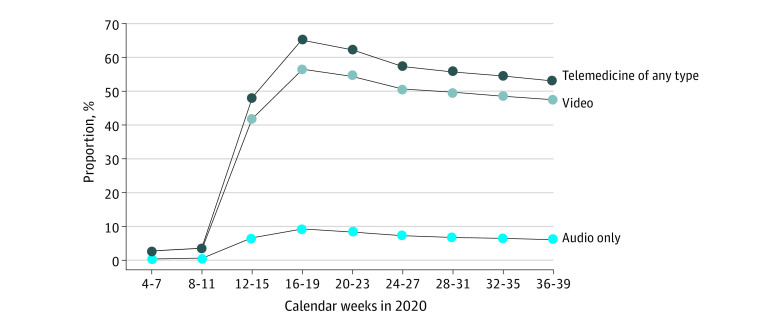
Proportion of Outpatient Mental Health Visits Conducted via Telemedicine in 2020

Telemedicine use for outpatient visits rapidly increased starting in weeks 12 to 15 of 2020, from 10 027 of 439 095 outpatient visits (2.3%) in weeks 4 to 7 of 2020 (prior to the pandemic), peaking at 255 201 of 391 457 visits (65.2%) in weeks 16 to 19 (ie, April 12 to May 9, 2020) and gradually declining to 213 867 of 403 048 (53.1%) by weeks 36 to 39 (Figure 1; eTable 3 in the Supplement). Video visits were the predominant form of telemedicine; audio-only visit use peaked in weeks 16 to 19 at 9.1% of all visits (35 687 of 391 457).

### Changes in Outpatient Visits

**Figure 2.**
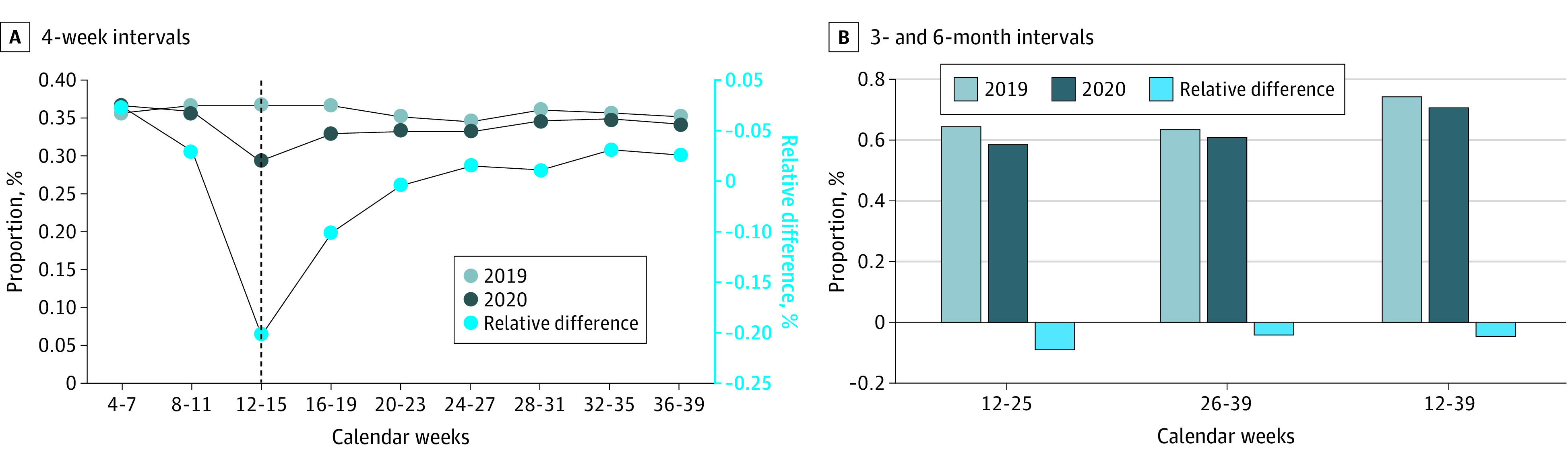
Differences in Outpatient Mental Health Utilization, 2019 vs 2020

In unadjusted analyses, there was a 20.3% relative decline in the fraction of individuals with an outpatient visit (telemedicine or in-person) in the first 4 weeks of the pandemic (200 590 [29.2%] in 2020 vs 265 169 [36.7%] in 2019), representing a 7.4 [95% CI, −7.6 to −7.3] percentage point decrease (Figure 2A; eTable 4 in the Supplement). In each subsequent 4-week period, the difference between 2020 and 2019 narrowed so that by weeks 36 to 39, the relative decrease was 2.5% (234 618 [34.2%] in 2020 vs 253 446 [35.1%] in 2019), a 0.9 (95% CI, −1.0 to −0.7) percentage point decrease. Changes in outpatient visits varied by outpatient treatment modality, with group therapy being the most disrupted initially and persistently through week 39 (eg, weeks 36-39: 11 275 [1.6%] in 2019 vs 5442 [0.8%] in 2020, a 0.77 [95% CI, −0.80 to −0.73] percentage point decrease and 49.4% relative decrease) (eTable 5 in the Supplement).

The fraction receiving any outpatient visits in the first and second 3-month periods of the pandemic also declined relative to the prior year (first 3 months: −5.9 [95% CI, −6.1 to −5.8] percentage point decrease, representing a 9.1% relative decrease; second 3 months: −2.6 [95% CI, −2.8 to −2.5] percentage point decrease, representing a 4.2% relative decrease) (Figure 2B; eTable 4 in the Supplement). During the entire 6-month pandemic period, 5.1% fewer individuals had any outpatient visits compared with the same period in 2019 (483 458 [70.5%] in 2020 vs 536 520 [74.2%] in 2019, a 3.8 [95% CI, −3.9 to −3.6] percentage point decrease).

In multivariable analyses, consistent with the bivariate findings, relative to the same weeks in 2019, the odds of having an outpatient mental health visit in the first and second 3 months of the pandemic were lower (weeks 12-25: odds ratio [OR], 0.78 [95% CI, 0.76-0.79]; weeks 26-39: OR, 0.92 [95% CI, 0.91-0.94]), as were the odds of receiving a visit over the entire 6-month pandemic period (OR, 0.84 [95% CI, 0.83-0.85]).

Independent of the pandemic, compared to White individuals, Black and Hispanic individuals had lower odds of receiving outpatient mental health visits (eg, weeks 12-25, Black individuals: OR, 0.75 [95% CI, 0.74-0.76]; Hispanic individuals: OR, 0.93 [95% CI, 0.91-0.95]), as did individuals dually eligible for Medicaid (OR, 0.72 [95% CI, 0.71-0.73]), rural residents (OR, 0.83 [95% CI, 0.82-0.84]), and those with a prior-year mental health hospitalization (OR, 0.87 [95% CI, 0.86-0.88]) or a medical condition increasing the risk of COVID-19 morbidity and mortality (OR, 0.89 [95% CI, 0.88-0.90]) (Table 2).

**Table 2.** Odds of Receiving an Outpatient Mental Health Visit After the Start of the COVID-19 Pandemic

Characteristic	OR (95% CI)^a^		
	Weeks 12-25	Weeks 26-39	Weeks 12-39
Main effects			
Year 2020 (vs 2019)	0.78 (0.76-0.79)	0.92 (0.91-00.94)	0.84 (0.83-0.85)
Race and ethnicity			
White	1 [Reference]	1 [Reference]	1 [Reference]
African American or Black	0.75 (0.74-0.76)	0.76 (0.75-0.77)	0.76 (0.74-0.77)
Hispanic or Latinx	0.93 (0.91-0.95)	0.95 (0.93-0.97)	0.95 (0.93-0.97)
Dually eligible for Medicaid	0.72 (0.71-0.73)	0.75 (0.74-0.76)	0.66 (0.66-0.67)
Disability	1.28 (1.26-1.30)	1.27 (1.25-1.29)	1.32 (1.30-1.34)
Rural	0.83 (0.82-0.84)	0.83 (0.82-0.84)	0.84 (0.83-0.85)
Comorbid SUD	1.09 (1.07-1.11)	1.08 (1.06-1.10)	1.15 (1.12-1.17)
MH/SUD hospitalization prior year	0.87 (0.86-0.88)	0.87 (0.86-0.88)	0.85 (0.83-0.86)
Medical comorbidity^b^	0.89 (0.88-0.90)	0.90 (0.89-0.91)	0.84 (0.83-0.85)
Differential changes in 2020			
Black × 2020^c^	0.99 (0.98-1.01)	0.97 (0.95-0.99)	0.97 (0.95-0.99)
Hispanic × 2020^c^	0.99 (0.96-1.01)	0.98 (0.95-1.01)	0.97 (0.95-1.00)
Dually eligible for Medicaid × 2020	1.00 (0.98-1.02)	0.96 (0.95-0.98)	1.00 (.98-1.02)
Disability × 2020	0.95 (0.93-0.96)	0.98 (0.97-0.998)	0.93 (0.92-0.95)
Rural × 2020	1.05 (1.04-1.07)	1.05 (1.04-1.06)	1.05 (1.04-1.07)
Comorbid SUD × 2020	1.07 (1.05-1.10)	1.05 (1.02-1.07)	1.04 (1.01-1.07)
MH hospitalization prior year × 2020	1.01 (0.99-1.03)	0.99 (0.97-1.01)	0.99 (0.97-1.01)
Medical comorbidity × 2020	1.05 (1.03-1.06)	1.00 (0.99-1.02)	1.04 (1.03-1.06)

Abbreviations: MH, mental health; OR, odds ratio; SUD, substance use disorder.

^a^ Regressions adjusted for age, documented sex, and US region in addition to other characteristics in Table 1, including race and ethnicity categories other (ie, Asian, American Indian or Alaska Native, and other) and unknown.

^b^ Any cardiovascular disease, hypertension, diabetes, or chronic lung disorder.

^c^ White patients are the reference category.

The differential decline in the odds of having an outpatient visit among those who had disability vs not was greater in both the first 3 months (OR, 0.95 [95% CI, 0.93-0.96]) and second 3 months (OR, 0.98 [95% CI, 0.97-0.998]) of the pandemic, relative to the same periods in 2019. During the second 3 months of the pandemic, relative to White individuals, Black individuals were differentially less likely to receive an outpatient mental health visit (weeks 26-39: OR, 0.97 [95% CI, 0.95-0.99]). This was echoed among those dually eligible for Medicaid (OR, 0.96 [95% CI, 0.95-0.98]). In contrast, rural residents or those with co-occurring substance use disorder had differentially greater odds of receiving an outpatient mental health visit in 2020 (eg, weeks 26-39, dual eligibility: OR, 1.05 [95% CI, 1.04-1.06]; co-occurring substance use disorder: OR, 1.05 [95% CI, 1.02-1.07]).

### Changes in Mental Health ED Visits, Inpatient Care, and Medication Use

**Figure 3.**
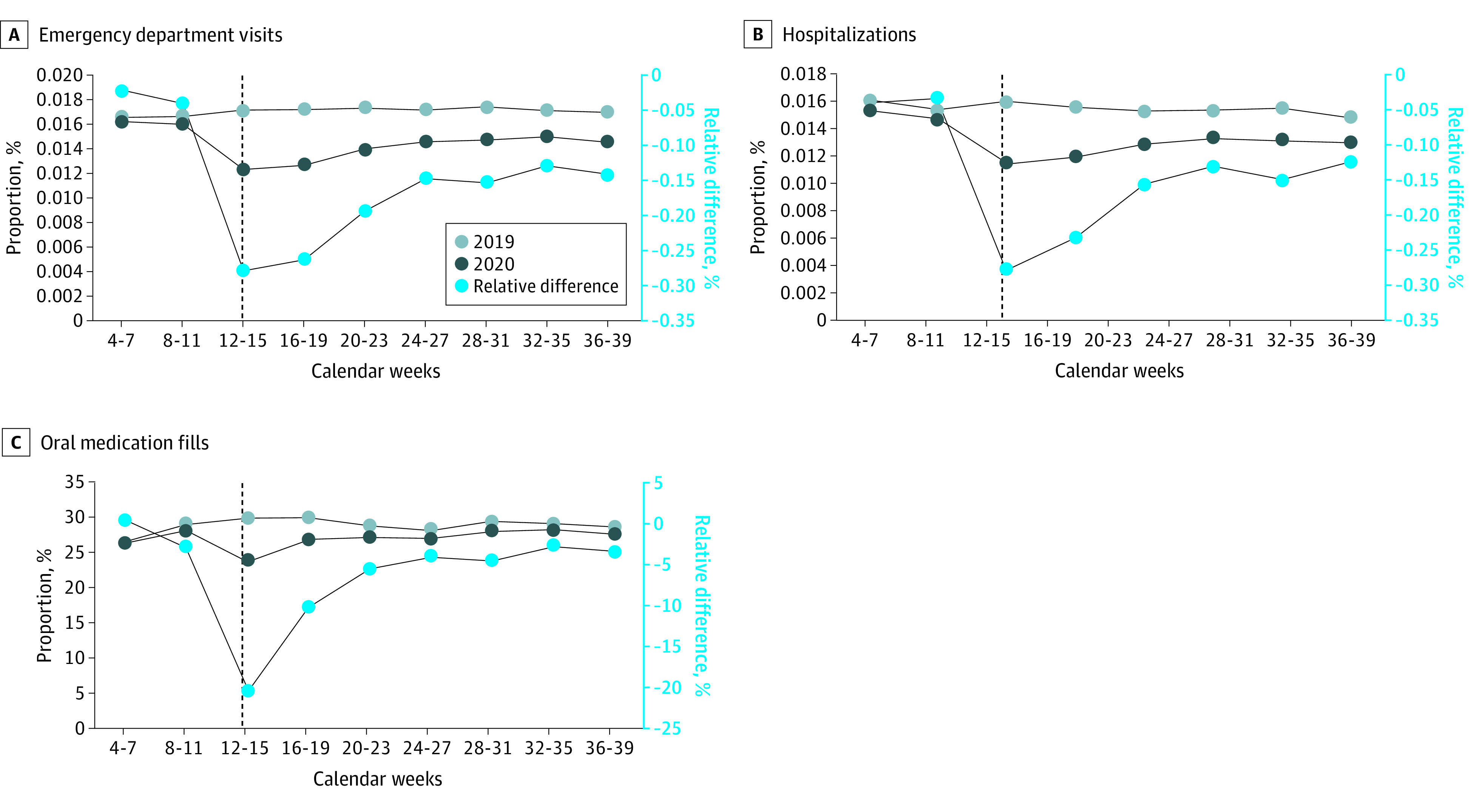
Differences in Mental Health Emergency Department, Hospitalization, and Medication Utilization, 2019 vs 2020

There was a 27.7% relative decrease in the fraction of individuals with an ED visit at the start of the pandemic (weeks 12-15) in 2020 (8503 [1.2%] in 2020 vs 12 383 [1.7%] in 2019, a 0.07 [95% CI, −0.11 to −0.02] percentage point decrease) (Figure 3; eTable 6 in the Supplement). This difference narrowed, and by weeks 36 to 39, the relative decrease was 14.1% (10 033 [1.5%] in 2020 vs 12 300 [1.7%] in 2019, a 0.24 [95% CI, −0.28 to −0.20] percentage point decrease).

Inpatient use also decreased, and reached its nadir (a 27.9% decrease), in weeks 12 to 15 of 2020 (7912 [1.2%] in 2020 vs 11 564 [1.6%] in 2019, a 0.36 [95% CI, −0.41 to −0.32] percentage point decrease). By weeks 32 to 35, inpatient admissions remained 12.4% lower (8933 [1.3%] in 2020 vs 10 750 [1.5%] in 2019, a 0.18 [95% CI, −0.22 to −0.15] percentage point decrease).

Similarly, the fraction of individuals with an oral medication prescription fill decreased most in the first weeks of the pandemic (20.3%) relative to the same weeks in 2019 (weeks 12-15: 163 796 [23.9%] in 2020 vs 216 468 [29.9%] in 2019, a 6.07 [95% CI, −6.21 to −5.92] percentage point decrease). By weeks 36 to 39, similar to outpatient visits, the fraction of patients with a prescription fill had largely rebounded but was still 3.4% lower (190 448 [27.8%] in 2020 vs 207 686 [28.7%] in 2019, a 0.97 [95% CI, −1.12 to −0.82] percentage point decrease).

## Discussion

In this national study of care among individuals with severe mental illness during the COVID-19 pandemic, we observed a sharp 20.3% decrease in outpatient mental health visit use and medication fills early in the pandemic when compared with the prior year that narrowed but remained (to approximately 2.5% and 3.4%, respectively) by the end of September 2020. The visit decrease was greater among Black individuals, those dually enrolled in Medicaid, and those with disability. Acute care utilization (ED, inpatient) among individuals with severe mental illness also decreased early in the pandemic (by 27.7% and 27.9%, respectively, in weeks 12-15) and remained lower through September 2020 (12.4% and 14.1%, respectively). Similar decreases in outpatient and acute care have been observed in other clinical populations during the pandemic—decreases thought to represent patients avoiding needed care rather than decreased acuity.^32,33^ Among individuals with psychotic disorders specifically, there are reports that the pandemic has been associated with increased symptom acuity.^34,35^ Thus, the decreased utilization we observed across all levels of care is even more concerning, even more so considering the high prevalence of disability (>80%) and poverty (>75%) in this study population.

Telemedicine visits were the majority of outpatient visits for patients with severe mental illness, particularly in the initial weeks of the pandemic, and were much higher than what has been observed in the Medicare population as a whole.^32^ Similarly, the early-pandemic decrease in outpatient visits we observed (20.3%) was not as steep as the decrease in office-based services in Medicare more generally (nearly 50%).^32,36^ While the disruptions in outpatient care we observed in this population are quite concerning, without telemedicine, the disruptions would likely have been much larger.

Later in the pandemic, we observed widened disparities in outpatient mental health care among those who were Black (relative to White) and Medicaid dual eligible (relative to nondual). While these differential changes were small in magnitude, they are concerning because they represent incremental worsening of sizable preexisting disparities.^16,17,18,19,20,37^ Other recent findings during the pandemic described worsening racial and ethnic disparities occurring in mental health care^38^ as well as in the health care of additional clinical populations.^39,40,41^ Future research will need to examine how these disparities evolve over time.

There are several potential reasons for the observed widening of outpatient care disparities. While many individuals with severe mental illness have access to smartphones,^5^ factors including homelessness or housing instability, isolation from support networks to troubleshoot technology, and both cognitive and mental health symptoms could contribute to a lowered ability to consistently connect to health care professionals via video telemedicine visit. The digital divide may more significantly affect populations with long-standing challenges accessing care, including Black and low-income populations.

### Limitations

Our study has several limitations. We identified severe mental illness using administrative data and not structured clinical interviews. However, claims data have demonstrated accuracy in establishing schizophrenia or bipolar disorder cohorts.^42,43,44^ The identified prevalence of substance use disorder in our cohorts (9%) was lower than one would expect based on prior literature,^45^ likely because substance use disorders are often underidentified in clinical care.^46,47^ Our results may not be generalizable to those insured by Medicaid or commercial insurance; compared with patients with serious mental illness insured by these other programs, individuals in our study population were more likely to have disability and be older. Additionally, among those dually eligible with Medicaid, we cannot observe utilization of Medicaid-covered services, such as assertive community treatment or intensive case management. We were unable to include long-acting injectable antipsychotics in the medication measure because of the imprecision of the claims data for this purpose.^48,49^ Also, we did not capture levels of symptoms or outcomes such as mortality. Prior reports indicate greater distress among individuals with psychotic disorders during the pandemic,^34,35^ and patients with severe mental illness are at higher risk of COVID-19–related mortality. Furthermore, we may have undercounted the number of outpatient visits conducted via telemedicine or undercounted the percentage conducted via audio only, particularly earlier in the pandemic when health care professionals who previously had not conducted telemedicine and were unfamiliar with the billing codes may have submitted claims as in-person or video visits.

## Conclusions

In this study, the first 6 months of the US COVID-19 emergency declaration were associated with large disruptions in mental health care for Medicare beneficiaries with severe mental illness. While disruptions peaked in the first month of the pandemic, outpatient visit use then rebounded with small but persistent reductions in care. Black patients and those with lower income experienced a larger decline in care, resulting in a widening disparity. Telemedicine was used for the majority of visits during the pandemic. Future work will be needed to understand the long-term consequences on health outcomes of these disruptions in care and how to address disparities in access to care for patients with severe mental illness.

## Notes

Supplement. **eFigure. **Flowchart for 2019 and 2020 Utilization Cohorts of Medicare Beneficiaries With Severe Mental Illness in Study **eTable 1. **Outpatient Visit Codes **eTable 2. **Substance Use Disorder, Medical Comorbidity, and Self-injury *ICD-10* Codes **eTable 3. **Weekly Number and Percentage of Outpatient Mental Health Visits in 2020 Conducted by Telemedicine and by Type of Telemedicine **eTable 4. **Outpatient Mental Health Utilization in 2019 vs 2020 **eTable 5. **Specific Types of Outpatient Mental Health Utilization in 2019 vs 2020 **eTable 6. **Mental Health Emergency Department Visit, Hospital Admission, or Oral Antipsychotic Medication Use in 2019 vs 2020

## References

[1] Kozloff N, Mulsant BH, Stergiopoulos V, Voineskos AN. The COVID-19 global pandemic: implications for people with schizophrenia and related disorders. Schizophr Bull. 2020;46(4):752-757. doi:10.1093/schbul/sbaa051 32343342 PMC7197583

[2] Shinn AK, Viron M. Perspectives on the COVID-19 pandemic and individuals with severe mental illness. J Clin Psychiatry. 2020;81(3):20com13412. doi:10.4088/JCP.20com13412 32369691

[3] Kasckow J, Felmet K, Appelt C, Thompson R, Rotondi A, Haas G. Telepsychiatry in the assessment and treatment of schizophrenia. Clin Schizophr Relat Psychoses. 2014;8(1):21-27A. doi:10.3371/CSRP.KAFE.021513 23428781 PMC4132656

[4] Santesteban-Echarri O, Piskulic D, Nyman RK, Addington J. Telehealth interventions for schizophrenia-spectrum disorders and clinical high-risk for psychosis individuals: a scoping review. J Telemed Telecare. 2020;26(1-2):14-20. doi:10.1177/1357633X18794100 30134781

[5] Young AS, Cohen AN, Niv N, . Mobile phone and smartphone use by people with serious mental illness. Psychiatr Serv. 2020;71(3):280-283. doi:10.1176/appi.ps.201900203 31744429 PMC7054173

[6] Patel SY, Mehrotra A, Huskamp HA, Uscher-Pines L, Ganguli I, Barnett ML. Variation in telemedicine use and outpatient care during the COVID-19 pandemic in the United States. Health Aff (Millwood). 2021;40(2):349-358. doi:10.1377/hlthaff.2020.01786 33523745 PMC7967498

[7] Raja PV, Gabrielian S, Doran N. Access to care for veterans with serious mental illness during the COVID-19 pandemic. Psychiatr Serv. 2021;72(11):1324-1327. doi:10.1176/appi.ps.202000898 34030456

[8] Wang B, Li R, Lu Z, Huang Y. Does comorbidity increase the risk of patients with COVID-19: evidence from meta-analysis. Aging (Albany NY). 2020;12(7):6049-6057. doi:10.18632/aging.103000 32267833 PMC7185114

[9] Zheng Z, Peng F, Xu B, . Risk factors of critical & mortal COVID-19 cases: a systematic literature review and meta-analysis. J Infect. 2020;81(2):e16-e25. doi:10.1016/j.jinf.2020.04.021 32335169 PMC7177098

[10] Nemani K, Li C, Olfson M, . Association of psychiatric disorders with mortality among patients with COVID-19. JAMA Psychiatry. 2021;78(4):380-386. doi:10.1001/jamapsychiatry.2020.4442 33502436 PMC7841576

[11] Lawrence D, Hancock KJ, Kisely S. The gap in life expectancy from preventable physical illness in psychiatric patients in Western Australia: retrospective analysis of population based registers. BMJ. 2013;346:f2539. doi:10.1136/bmj.f2539 23694688 PMC3660620

[12] Brown S, Kim M, Mitchell C, Inskip H. Twenty-five year mortality of a community cohort with schizophrenia. Br J Psychiatry. 2010;196(2):116-121. doi:10.1192/bjp.bp.109.067512 20118455 PMC4560167

[13] Sharma M, Lioutas V-A, Madsen T, . Decline in stroke alerts and hospitalisations during the COVID-19 pandemic. Stroke Vasc Neurol. 2020;5(4):403-405. doi:10.1136/svn-2020-000441 32855352 PMC7453762

[14] Wong LE, Hawkins JE, Langness S, Murrell KL, Iris P, Sammann A. Where are all the patients? addressing COVID-19 fear to encourage sick patients to seek emergency care. NEJM Catalyst. May 14, 2020. Accessed December 22, 2021. https://catalyst.nejm.org/doi/full/10.1056/CAT.20.0193

[15] Bhatt AS, Moscone A, McElrath EE, . Fewer hospitalizations for acute cardiovascular conditions during the COVID-19 pandemic. J Am Coll Cardiol. 2020;76(3):280-288. doi:10.1016/j.jacc.2020.05.038 32470516 PMC7250561

[16] 2018 National healthcare quality and disparities Report. Agency for Healthcare Research and Quality, Reviewed April 2020. Accessed December 22, 2021. https://www.ahrq.gov/research/findings/nhqrdr/nhqdr18/index.html

[17] Ault-Brutus AA. Changes in racial-ethnic disparities in use and adequacy of mental health care in the United States, 1990–2003. Psychiatr Serv. 2012;63(6):531-540. doi:10.1176/appi.ps.201000397 22422014

[18] Busch AB, Huskamp HA, Neelon B, Manning T, Normand S-LT, McGuire TG. Longitudinal racial/ethnic disparities in antimanic medication use in bipolar-I disorder. Med Care. 2009;47(12):1217-1228. doi:10.1097/MLR.0b013e3181adcc4f 19786909 PMC2787883

[19] Busch AB, Lehman AF, Goldman H, Frank RG. Changes over time and disparities in schizophrenia treatment quality. Med Care. 2009;47(2):199-207. doi:10.1097/MLR.0b013e31818475b7 19169121 PMC2789766

[20] Phillips KL, Copeland LA, Zeber JE, Stock EM, Tsan JY, MacCarthy AA. Racial/ethnic disparities in monitoring metabolic parameters for patients with schizophrenia receiving antipsychotic medications. Am J Geriatr Psychiatry. 2015;23(6):596-606. doi:10.1016/j.jagp.2014.07.007 25154537

[21] Stockdale SE, Lagomasino IT, Siddique J, McGuire T, Miranda J. Racial and ethnic disparities in detection and treatment of depression and anxiety among psychiatric and primary health care visits, 1995-2005. Med Care. 2008;46(7):668-677. doi:10.1097/MLR.0b013e3181789496 18580385 PMC3956700

[22] Ziller EC, Anderson NJ, Coburn AF. Access to rural mental health services: service use and out-of-pocket costs. J Rural Health. 2010;26(3):214-224. doi:10.1111/j.1748-0361.2010.00291.x 20633089

[23] Thomas D, Macdowell M, Glasser M. Rural mental health workforce needs assessment—a national survey. Rural Remote Health. 2012;12:2176. doi:10.22605/RRH2176 23088609

[24] Kopec K, Janney CA, Johnson B, Spykerman K, Ryskamp B, Achtyes ED. Rapid transition to telehealth in a community mental health service provider during the COVID-19 pandemic. Prim Care Companion CNS Disord. 2020;22(5):20br02787. doi:10.4088/PCC.20br02787 33002350

[25] Khaykin E, Eaton WW, Ford DE, Anthony CB, Daumit GL. Health insurance coverage among persons with schizophrenia in the United States. Psychiatr Serv. 2010;61(8):830-834. doi:10.1176/ps.2010.61.8.830 20675843 PMC7245045

[26] von Elm E, Altman DG, Egger M, Pocock SJ, Gøtzsche PC, Vandenbroucke JP; STROBE Initiative. The Strengthening the Reporting of Observational Studies in Epidemiology (STROBE) statement: guidelines for reporting observational studies. PLoS Med. 2007;4(10):e296. doi:10.1371/journal.pmed.0040296 17941714 PMC2020495

[27] Busch AB, Frank RG, Lehman AF. The effect of a managed behavioral health carve-out on quality of care for Medicaid patients diagnosed as having schizophrenia. Arch Gen Psychiatry. 2004;61(5):442-448. doi:10.1001/archpsyc.61.5.442 15123488

[28] Fullerton CA, Busch AB, Frank RG. The rise and fall of gabapentin for bipolar disorder: a case study on off-label pharmaceutical diffusion. Med Care. 2010;48(4):372-379. doi:10.1097/MLR.0b013e3181ca404e 20195173 PMC4879613

[29] Fung V, Price M, Busch AB, Dow W, Fireman B, Hui R, . Medicare Part D cost-sharing and antipsychotic drug use in two Medicare Advantage systems. Clin Med Res. 2012;10(3):182. doi:10.3121/cmr.2012.1100.cb7-02

[30] Hedegaard H, Schoenbaum M, Claassen C, Crosby A, Holland K, Proescholdbell S. Issues in developing a surveillance case definition for nonfatal suicide attempt and intentional self-harm using *International Classification of Diseases, Tenth Revision, Clinical Modification* (*ICD-10-CM*) coded data. Natl Health Stat Report. 2018;(108):1-19. 29616901

[31] Medicare Learning Network Fact Sheet. Telehealth services. Accessed December 22, 2021. https://www.cms.gov/Outreach-and-Education/Medicare-Learning-Network-MLN/MLNProducts/Downloads/TelehealthSrvcsfctsht.pdf

[32] Bosworth A, Ruhter J, Samson LW, Sheingold S, Taplin C, Tarazi W, . Medicare beneficiary use of telehealth visits: early data from the start of the COVID-19 pandemic. Office of the Assistant Secretary for Planning and Evaluation. July 28, 2020. Accessed December 22, 2021. https://aspe.hhs.gov/reports/medicare-beneficiary-use-telehealth-visits-early-data-start-covid-19-pandemic

[33] Solomon MD, McNulty EJ, Rana JS, . The COVID-19 pandemic and the incidence of acute myocardial infarction. N Engl J Med. 2020;383(7):691-693. doi:10.1056/NEJMc2015630 32427432

[34] Abbas MJ, Kronenberg G, McBride M, . The early impact of the COVID-19 pandemic on acute care mental health services. Psychiatr Serv. 2021;72(3):242-246. doi:10.1176/appi.ps.202000467 33076794

[35] Szmulewicz AG, Benson NM, Hsu J, Hernán MA, Öngür D. Effects of COVID-19 pandemic on mental health outcomes in a cohort of early psychosis patients. Early Interv Psychiatry. 2021;15(6):1799-1802. doi:10.1111/eip.13113 33432786 PMC8013468

[36] Bosworth A, Ruhter J, Sheingold S, Zuckerman R. The impact of the COVID-19 pandemic on Medicare beneficiary use of health care services and payments to providers: early data for the first 6 months of 2020. Assistant Secretary for Planning and Evaluation. September 28, 2020. Accessed December 22, 2021. https://aspe.hhs.gov/sites/default/files/private/pdf/264071/Medicare-FFS-Spending-Utilization.pdf

[37] Heun-Johnson H, Menchine M, Axeen S, . Association between race/ethnicity and disparities in health care use before first-episode psychosis among privately insured young patients. JAMA Psychiatry. 2021;78(3):311-319. doi:10.1001/jamapsychiatry.2020.3995 33355626 PMC7758828

[38] Yang J, Landrum MB, Zhou L, Busch AB. Disparities in outpatient visits for mental health and/or substance use disorders during the COVID surge and partial reopening in Massachusetts. Gen Hosp Psychiatry. 2020;67:100-106. doi:10.1016/j.genhosppsych.2020.09.004 33091782 PMC7550185

[39] Khatana SAM, Groeneveld PW. Health disparities and the coronavirus disease 2019 (COVID-19) pandemic in the USA. J Gen Intern Med. 2020;35(8):2431-2432. doi:10.1007/s11606-020-05916-w 32462564 PMC7251802

[40] Lieberman-Cribbin W, Tuminello S, Flores RM, Taioli E. Disparities in COVID-19 testing and positivity in New York City. Am J Prev Med. 2020;59(3):326-332. doi:10.1016/j.amepre.2020.06.005 32703702 PMC7316038

[41] Shah GH, Shankar P, Schwind JS, Sittaramane V. The detrimental impact of the COVID-19 crisis on health equity and social determinants of health. J Public Health Manag Pract. 2020;26(4):317-319. doi:10.1097/PHH.0000000000001200 32433385

[42] Lurie N, Popkin M, Dysken M, Moscovice I, Finch M. Accuracy of diagnoses of schizophrenia in Medicaid claims. Hosp Community Psychiatry. 1992;43(1):69-71. doi:10.1176/ps.43.1.69 1544654

[43] Unützer J, Simon G, Pabiniak C, Bond K, Katon W. The treated prevalence of bipolar disorder in a large staff-model HMO. Psychiatr Serv. 1998;49(8):1072-1078. doi:10.1176/ps.49.8.1072 9712215

[44] Unützer J, Simon G, Pabiniak C, Bond K, Katon W. The use of administrative data to assess quality of care for bipolar disorder in a large staff model HMO. Gen Hosp Psychiatry. 2000;22(1):1-10. doi:10.1016/S0163-8343(99)00057-2 10715498

[45] Hunt GE, Large MM, Cleary M, Lai HMX, Saunders JB. Prevalence of comorbid substance use in schizophrenia spectrum disorders in community and clinical settings, 1990-2017: systematic review and meta-analysis. Drug Alcohol Depend. 2018;191:234-258. doi:10.1016/j.drugalcdep.2018.07.011 30153606

[46] Condren RM, O'Connor J, Browne R. Prevalence and patterns of substance misuse in schizophrenia: a catchment area case-control study. Psychiatric Bulletin. 2018;25(1):17-20. doi:10.1192/pb.25.1.17

[47] Ley A, Jeffery D, Ruiz J, McLaren S, Gillespie C. Underdetection of comorbid drug use at acute psychiatric admission. Psychiatric Bulletin. 2018;26(7):248-251. doi:10.1192/pb.26.7.248

[48] Parellada E, Bioque M. Barriers to the use of long-acting injectable antipsychotics in the management of schizophrenia. CNS Drugs. 2016;30(8):689-701. doi:10.1007/s40263-016-0350-7 27255405

[49] West JC, Marcus SC, Wilk J, Countis LM, Regier DA, Olfson M. Use of depot antipsychotic medications for medication nonadherence in schizophrenia. Schizophr Bull. 2008;34(5):995-1001. doi:10.1093/schbul/sbm137 18093962 PMC2518642

