# Supplementary material for: Disruptions in Care for Medicare Beneficiaries With Severe Mental Illness During the COVID-19 Pandemic
Source: JAMA Netw Open. 2022 Jan 28;5(1):e2145677. doi: 10.1001/jamanetworkopen.2021.45677 (PMC8800078; doi:10.1001/jamanetworkopen.2021.45677)
Supplement: Supplement. — eFigure. Flowchart for 2019 and 2020 Utilization Cohorts of Medicare Beneficiaries With Severe Mental Illness in Study eTable 1. Outpatient Visit Codes eTable 2. Substance Use Disorder, Medical Comorbidity, and Self-injury ICD-10 Codes eTable 3. Weekly Number and Percentage of Outpatient Mental Health Visits in 2020 Conducted by Telemedicine and by Type of Telemedicine eTable 4. Outpatient Mental Health Utilization in 2019 vs 2020 eTable 5. Specific Types of Outpatient Mental Health Utilization in 2019 vs 2020 eTable 6. Mental Health Emergency Department Visit, Hospital Admission, or Oral Antipsychotic Medication Use in 2019 vs 2020 [file jamanetwopen-e2145677-s001.pdf]

Supplemental Online Content

Busch AB, Huskamp HA, Raja P, Rose S, Mehrotra A. Disruptions in care for Medicare beneficiaries with severe mental illness during the COVID-19 pandemic. *JAMA Netw Open.* 2022;5(1):e2145677. doi:10.1001/jamanetworkopen.2021.45677

eFigure. Flowchart for 2019 and 2020 Utilization Cohorts of Medicare Beneficiaries With Severe Mental Illness in Study

eTable 1. Outpatient Visit Codes

eTable 2. Substance Use Disorder, Medical Comorbidity, and Self-injury *ICD-10* Codes

eTable 3. Weekly Number and Percentage of Outpatient Mental Health Visits in 2020 Conducted by Telemedicine and by Type of Telemedicine

eTable 4. Outpatient Mental Health Utilization in 2019 vs 2020

eTable 5. Specific Types of Outpatient Mental Health Utilization in 2019 vs 2020

eTable 6. Mental Health Emergency Department Visit, Hospital Admission, or Oral Antipsychotic Medication Use in 2019 vs 2020

This supplemental material has been provided by the authors to give readers additional information about their work.

© 2022 Busch AB et al. *JAMA Network Open.*

eFigure. Flowchart for 2019 and 2020 Utilization Cohorts of Medicare Beneficiaries With Severe Mental Illness in Study

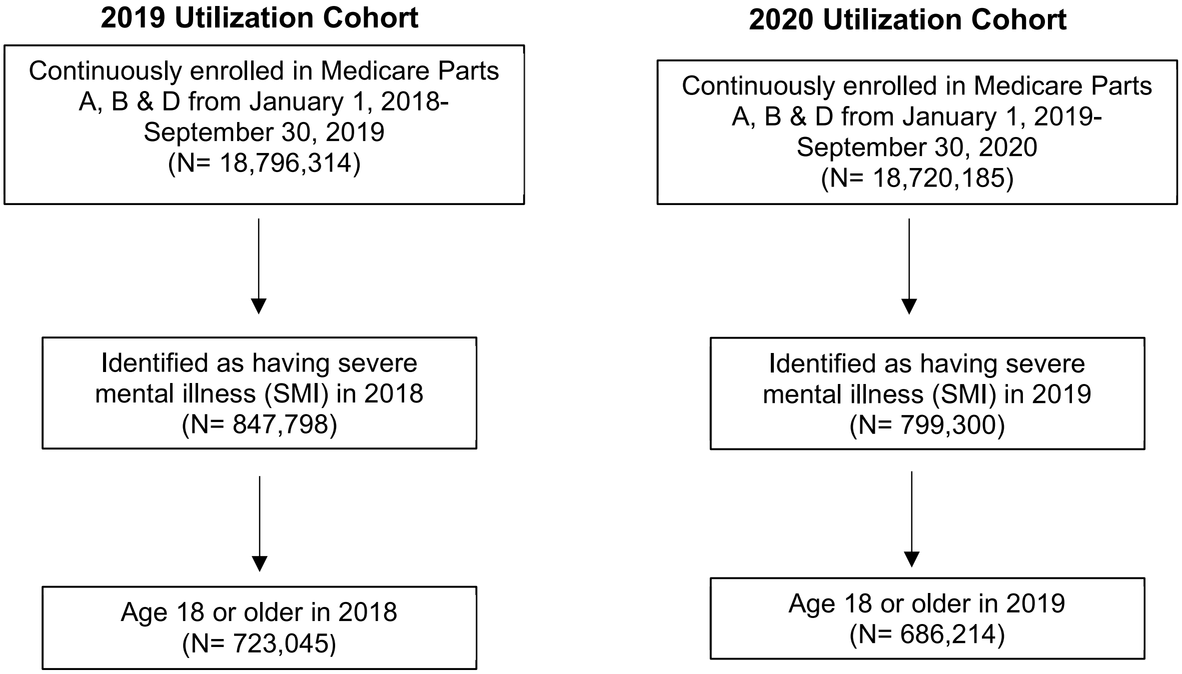

© 2022 Busch AB et al. *JAMA Network Open.*

eTable 1. Outpatient Visit Codes

	MH/SUD Services Category	CPT/HCPCS Codes
Outpatient Codes	ECT or TMS services	90867-90871
	Crisis intervention services	90839, 90840, H0007, H2011, S9484, S9485, T2034
	Assessments, E&M services, substance use disorders medication services	90791, 90792, 99058, 99201-99205, 99211-99215, 99241-99245, 99341-99345, 99347, 99350, 99495, 99496, G0155, G0175, G0463, G0466, G0467, G0469, G0470, H0001, H0002, H0014, H0016, H0020, H0022, H0023, H0031, H0034, H0038, H0046, H1011, H2000, H2010, H2027, H5030, M0064, T1007, T1011, T1015, T0123, T1040, T1041, T2010, T2011, Z0001
	Psychotherapy services	Individual or Family: 90832-90838, 90845, 90847-90849, 90865, 90875, 90876, 90880, 90900-90902, 90904, 90906, 90908, 90910, 99510, H0004, H2019, H2020, H2032, H5010, T1006, T1012<br>Group: 90853, 90857, H0005, H5020, H5025, S9454,
	Intensive outpatient services	H0015, S9480
	Supportive psychosocial services	97003, 97004, 99490, H0036, H0037, H0039, H0040, H2001, H2013-H2018, H2021-H2026, H2030, H2031, H5220, H5230, H5240, H5299, S9127, T1017, T2012-T2015, T2018-T2023, Z0002,
	Screening and preventative counseling or services	98960-98962, 99078, 99385-99387, 99395-99397, 99401-99404, 99408, 99409, 99411, 99412, 99420, G0396, G0397, G0442, G0443, G0513-G0515, H0028, H0029, H0049, H0050.
	Codes specific for telemedicine visits	Audio only: 99441-99443, 98966-98968<br>Video: G2025 or outpatient visit with modifier code (modifiers GQ, GT or 95)
Emergency Department Codes		90500, 90510, 90515, 90517, 90520, 90530, 90540, 90550, 90560, 90570, 90580, 99281-99285

© 2022 Busch AB et al. *JAMA Network Open.*

eTable 2. Substance Use Disorder, Medical Comorbidity, and Self-injury *ICD-10* Codes

Diagnosis	ICD-10 Codes
Intentional self-harm	R45.851, X71-X83, T14.91, T36-T65 and T71 when 6th character of code =2.<br>Except for T36.9, T37.9, T39.9, T41.4, T42.7, T43.9, T45.9, T47.9, and T49.9, T51.9, T52.9, T53.9, T54.9, T56.9, T57.9, T58.0, T58.1, T58.9, T59.9, T60.9, T61.0, T61.1, T61.9, T62.9, T63.9, T64.0, T64.8, T65.9, which are included if the 5th character of the code=2.
Accidental or unclear intent overdose/poisoning by substances of abuse	R78.0 (excessive blood alcohol level)<br>OR<br>T40.0 (opium), T40.1 (heroin), T40.2 (other opioids), T40.3 (methadone), T40.4 (synthetic narcotics), T40.5 (cocaine)<br>T40.6 (unspecified narcotic), T40.7 (cannabis/derivatives), T40.8 (LSD), T40.90 (unspecified hallucinogens), T40.99 (other hallucinogens), T42.3 (barbiturates), T42.4 (benzodiazepines), T43.60 (psychostimulants), T43.62 (amphetamines), T43.63 (methylphenidate), T43.64 (ecstasy), or T43.69 (other psychostimulants), T51.0X<br>when 6th character =1 or 4
Substance use disorder	F10-F16 or F18-F19
Diabetes	E08.xxx-E11.xxx, E13.xxx
Cardiovascular disease	Heart failure: I09.81, I11.0, I13.0, I13.2, I50.1, I50.2x, I50.3x, I50.4x, I50.81x, I50.82, I50.83, I50.84, I50.89, I50.9<br>**OR**<br>Ischemic heart disease: I20.xxx-I25.xxx
Hypertension	H35.031, H35.032, H35.033, H35.039, I10, I11.0, I11.9, I12.0, I12.9, I13.0, I13.1x, I13.2, I15.x, I67.4, N26.2
Chronic lung problems	Asthma: J45.2xx-J45.5xx, J45.9xx<br>**OR**<br>COPD: J40.xx-J44.xx, J47.xx

© 2022 Busch AB et al. *JAMA Network Open.*

eTable 3. Weekly Number and Percentage of Outpatient Mental Health Visits in 2020 Conducted by Telemedicine and by Type of Telemedicine

Calendar Weeks in 2020	N of Outpatient Visits (in-person and telemedicine)	Mental health telemedicine visit (any type)		Mental health telemedicine video visit		Mental health telemedicine audio only visit	
		N	% of outpatient visits	N	% of outpatient visits	N	% of outpatient visits
4-7	439,095	10,027	2.3	9,988	2.3	50	0.01
8-11	431,126	14,445	3.4	14,334	3.3	129	0.03
12-15	334,880	160,187	47.8	139,797	41.7	20,960	6.3
16-19	391,457	255,201	65.2	220,513	56.3	35,687	9.1
20-23	395,911	247,011	62.4	215,963	54.5	32,193	8.1
24-27	393,963	225,864	57.3	199,225	50.6	27,799	7.1
28-31	411,259	229,262	55.8	203,464	49.5	27,014	6.6
32-35	413,035	225,011	54.5	200,204	48.5	25,958	6.3

Notes: (1) Unadjusted analyses. (2) Federal COVID-10 pandemic emergency declaration began week 12 of 2020.

eTable 4. Outpatient Mental Health Utilization in 2019 vs 2020

	**2019 N=723045**		**2020 N=686214**		**Percentage point difference (95% CI)**	**% Relative change in 2020 compared to 2019**
	**N**	**%**	**N**	**%**		
Week 4-7	257,782	35.7	250,350	36.5	0.8 (0.7, 1.0)	2.3
Week 8-11	264,197	36.5	245,723	35.8	-0.7 (-0.9, -0.6)	-2.0
Weeks 12-15	265,169	36.7	200,590	29.2	-7.4 (-7.6, -7.3)	-20.3
Weeks 16-19	264,819	36.6	225,825	32.9	-3.7 (-3.9, -3.6)	-10.2
Weeks 20-23	254,550	35.2	228,525	33.3	-1.9 (-2.1, -1.8)	-5.4
Weeks 24-27	249,158	34.5	228,074	33.2	-1.2 (-1.4, -1.1)	-3.6
Weeks 28-31	260,667	36.1	237,558	34.6	-1.4 (-1.6, -1.3)	-4.0
Weeks 32-35	257,311	35.6	239,492	34.9	-0.7 (-84. -0.53)	-1.9
Weeks 36-39	253,446	35.1	234,618	34.2	-0.9 (-1.0, -0.7)	-2.5
**Weeks 12-25**	**466,379**	**64.5**	**402,065**	**58.6**	**-5.9 (-6.1, - 5.8)**	**-9.1**
**Weeks 26-39**	**456,048**	**63.1**	**414,752**	**60.4**	**-2.6 (-2.8, -2.5)**	**-4.2**
**Weeks 12-39**	**536,520**	**74.2**	**483,458**	**70.5**	**-3.8 (-3.9, -3.6)**	**-5.1**

Notes: (1) Having at least one outpatient mental health visit during the observation interval. (2) Unadjusted analyses. (3) Week 1 in 2020 is one day shorter than week 1 in 2019 due to January 1st 2019 vs. 2020 start dates. Therefore, estimates begin with weeks 4-7.

© 2022 Busch AB et al. *JAMA Network Open.*

eTable 5. Specific Types of Outpatient Mental Health Utilization in 2019 vs 2020

	2019 N=723045		2020 N=686214		Percentage Point Difference (95% CI)	% Relative change in 2020 compared to 2019
**Individual or Family therapy**	**N**	**%**	**N**	**%**		
Week 4-7	111,156	15.37	108,139	15.76	0.39 (-0.51, -0.27)	2.5
Week 8-11	114,385	15.82	107,803	15.71	-0.11 (-0.23, 0.01)	-0.7
Weeks 12-15	116,410	16.10	88,210	12.85	-3.25 (-3.36, -3.13)	-20.2
Weeks 16-19	115,419	15.96	93,474	13.62	-2.34 (-2.46, -2.22)	-14.7
Weeks 20-23	111,680	15.45	95,189	13.87	-1.57 (-1.69, -1.46)	-10.2
Weeks 24-27	109,645	15.16	95,226	13.88	-1.29 (-1.40, -1.17)	-8.4
Weeks 28-31	112,720	15.59	97,246	14.17	-1.42 (-1.54, -1.30)	-9.1
Weeks 32-35	112,043	15.50	100,301	14.62	-.88 (-1.00, -0.76)	-5.7
Weeks 36-39	111,080	15.36	97,707	14.24	-1.12 (-1.24, -1.01)	-7.3
**Group therapy**	**N**	**%**	**N**	**%**		
Week 4-7	12,025	1.66	10,905	1.59	-0.07 (-0.12. -0.03)	-4.2
Week 8-11	12,288	1.70	10,618	1.55	-0.15 (-0.19, -0.11)	-8.8
Weeks 12-15	12,400	1.71	3,823	0.56	-1.16 (-1.19, -1.12)	-67.3
Weeks 16-19	12,007	1.66	3,446	0.50	-1.16 (-1.19, -1.12)	-69.9
Weeks 20-23	11,396	1.58	4,135	0.60	-0.97 (-1.01, -0.94)	-62.0
Weeks 24-27	11,080	1.53	4,324	0.63	-0.90 (-0.94, -0.87)	-58.8
Weeks 28-31	10,777	1.49	4,153	0.61	-0.89 (-0.92, -0.85)	-59.1
Weeks 32-35	9,397	1.30	4,868	0.71	-0.59 (-0.62, -0.59)	-45.4
Weeks 36-39	11,275	1.56	5,442	0.79	-0.77 (-0.80. -0.73)	-49.4
**ECT**	**N**	**%**	**N**	**%**		
Week 4-7	1,546	0.21	1,475	0.21	0.001 (-0.014, 0.016)	0.0
Week 8-11	1,572	0.22	1,472	0.21	-0.003 (-0.018, -0.012)	-4.5
Weeks 12-15	1,542	0.21	933	0.14	-0.08 (-0.09, -0.06)	-33.3
Weeks 16-19	1,537	0.21	890	0.13	-0.08 (-0.10, -0.07)	-38.1
Weeks 20-23	1,482	0.20	1,069	0.16	-0.05 (-0.06, -0.04)	-20.0
Weeks 24-27	1,491	0.21	1,103	0.16	-0.05 (-0.06, -0.03)	-23.8
Weeks 28-31	1,465	0.20	1,152	0.17	-0.03 (-0.05, -0.02)	-15.0
Weeks 32-35	1,491	0.21	1,175	0.17	-0.03 0.05, -0.02)	-19.0
Weeks 36-39	1,499	0.21	1,151	0.17	-0.04 (-0.05, -0.03)	-19.0

Notes: (1) Having at least one outpatient mental health visit (by type) during the observation interval. (2) Unadjusted analyses. (3) Week 1 in 2020 is one day shorter than week 1 in 2019 due to January 1st 2019 vs. 2020 start dates. Therefore, estimates begin with weeks 4-7.

© 2022 Busch AB et al. *JAMA Network Open.*

eTable 6. Mental Health Emergency Department Visit, Hospital Admission, or Oral Antipsychotic Medication Use in 2019 vs 2020

	2019 N=723045		2020 N=686214		Percentage Point Difference (95% CI)	% Relative change in 2020 compared to 2019
**ED Visit**	**N**	**%**	**N**	**%**		
Week 4-7	11969	1.7	11102	1.6	-0.04 (-0.08, -0.00)	-2.3
Week 8-11	12063	1.7	11001	1.6	-0.07 (-0.11, -0.02)	-3.9
Weeks 12-15	12383	1.7	8503	1.2	-0.47 (-0.51, -0.43)	-27.6
Weeks 16-19	12440	1.7	8710	1.3	-0.45 (-0.49, -0.41)	-26.2
Weeks 20-23	12545	1.7	9601	1.4	-0.34 (-0.38, 0.-29)	-19.4
Weeks 24-27	12390	1.7	10031	1.5	-0.25 (-0.29, -0.21)	-14.7
Weeks 28-31	12591	1.7	10134	1.5	-0.26 (-0.31, -0.22)	-15.2
Weeks 32-35	12428	1.7	10274	1.5	-0.22 (-0.26, -0.18)	-12.9
Weeks 36-39	12300	1.7	10033	1.5	-0.24 (-0.28, -0.20)	-14.1
**Hospitalization**	**N**	**%**	**N**	**%**		
Week 4-7	11630	1.6	10573	1.5	-0.07 (-0.11, -0.03)	-4.2
Week 8-11	11063	1.5	10142	1.5	0-.05 (-0.09, -0.01)	-3.4
Weeks 12-15	11564	1.6	7912	1.2	-0.45 (-0.48, -0.41)	-27.9
Weeks 16-19	11244	1.6	8204	1.2	-0.36 (-0.41, -0.32)	-23.1
Weeks 20-23	11063	1.5	8854	1.3	-0.24 (-0.28, -0.20)	-15.7
Weeks 24-27	11121	1.5	9173	1.3	-0.20 (-0.24, -0.16)	-13.1
Weeks 28-31	11227	1.6	9064	1.3	-0.23 (-0.27, -0.19)	-14.9
Weeks 32-35	10750	1.5	8933	1.3	-0.18 (-0.22, -0.15)	-12.4
**Oral Antipsychotic Medications**	**N**	**%**	**N**	**%**		
Week 4-7	190,501	26.3	181,631	26.5	0.12 (-0.27, 0.02)	0.5
Week 8-11	210,472	29.1	194,248	28.3	-0.80 (-0.95, -0.65)	-2.8
Weeks 12-15	216,468	29.9	163,796	23.9	-6.07 (-6.21, -5.92)	-20.3
Weeks 16-19	217,156	30.0	185,346	27.0	-3.02 (-3.17, -2.87)	-10.1
Weeks 20-23	208,812	28.9	187,223	27.3	-1.60 (-1.74, -1.45)	-5.5
Weeks 24-27	204,272	28.3	186,168	27.1	-1.12 (-1.27, -0.97)	-4.0
Weeks 28-31	213,141	29.5	193,222	28.2	-1.32 (-1.47, -1.17)	-4.5
Weeks 32-35	211,044	29.2	194,913	28.4	-0.78 (-0.93, -0.63)	-2.7
Weeks 36-39	207,686	28.7	190,448	27.8	-0.97 (-1.12, -0.82)	-3.4

Notes: (1) Having at least one outpatient mental health visit during the observation interval. (2) Unadjusted analyses. (3) Week 1 in 2020 is one day shorter than week 1 in 2019 due to January 1st 2019 vs. 2020 start dates. Therefore, estimates begin with weeks 4-7.

© 2022 Busch AB et al. *JAMA Network Open.*
